# Pain and Frailty in Childhood Cancer Survivors: A Narrative Review

**DOI:** 10.3390/curroncol32010022

**Published:** 2024-12-31

**Authors:** Chiara Papini, Jaspreet K. Sodhi, Cassie M. Argenbright, Kirsten K. Ness, Tara M. Brinkman

**Affiliations:** 1 Psychology and Biobehavioral Sciences, St. Jude Children’s Research Hospital, 262 Danny Thomas Place, Memphis, TN 38105, USA; 2 School of Physical Therapy, Marshall University, 2847 5th Ave, Huntington, WV 25702, USA; 3 Epidemiology and Cancer Control, St. Jude Children’s Research Hospital, 262 Danny Thomas Place, Memphis, TN 38105, USA

**Keywords:** pain, frailty, childhood cancer survivors

## Abstract

A significant proportion of childhood cancer survivors experience persistent health problems related to cancer or cancer treatment exposures, including accelerated or early onset of aging. Survivors are more likely than non-cancer peers to present a frail phenotype suggestive of reduced physiologic reserve and have symptoms that interfere with function in daily life, including pain. Studies in the general population, mostly among older adults, suggest that pain is a significant contributor to development and progression of frail health. This association has not been explored among childhood cancer survivors. In this narrative review, we highlight this gap by summarizing the epidemiologic evidence on pain and frailty, including their prevalence, common risk factors, and correlates in childhood cancer survivors. We further discuss associations between pain and frailty in non-cancer populations, likely biological mechanisms in survivors, and potential interventions targeting both domains.

## 1. Introduction

Advances in cancer care and treatment among children with cancer have markedly improved survival. Over 85% of children diagnosed with cancer will survive five years, with an estimated 500,000 survivors of childhood cancer living in the United States today [1]. However, this success is not without substantial cost for survivors. Treatment-related consequences and late effects are prevalent [2,3,4,5]. Survivors have acute problems that can persist and are at increased risk for developing chronic disease [6,7,8,9]. Even those who do not have clinically overt chronic disease report symptoms that interfere with function and impact participation in daily life [10,11]. One of the most common symptoms reported by children during cancer therapy is pain [12,13]. Pain is also prevalent in long-term survivors of childhood cancer [14], interferes with performance of daily activities [15], limits participation in social roles [15], and affects quality of life [16,17]. Pain can be disabling because it limits movement, likely contributing to functional limitations seen in young adult survivors of childhood cancer [4] and perpetuating the early aging process observed in this population [18]. Like older adults, childhood cancer survivors are at increased risk for frail health, a phenotype characterized by reduced physiologic reserve [18,19]. Among older adults without cancer, pain is associated with frailty [20] and increases susceptibility to chronic diseases [21], falls [22], hospitalization [23], and mortality [24].

Although reports of pain and frailty in long-term survivors of childhood cancer are emerging [25], limited data are available describing associations between pain and either frail health or subsequent disability. While other recent reviews have focused separately on pain [26,27] or frailty [28,29] in childhood cancer survivors, in this review we aim to provide (1) a summary of the epidemiologic evidence about pain and frailty in childhood cancer survivors, including their prevalence and common determinants; (2) an overview of the potential associations between pain and frailty based on non-cancer populations; and (3) a discussion of potential interventions to treat pain and frailty among childhood cancer survivors.

## 2. Materials and Methods

An initial literature search on PubMed, PsycInfo, and Google Scholar was conducted using the key word ‘childhood cancer survivors’ and each of the following: ‘pain’, ‘frailty’, ‘emotional health’, ‘chronic conditions’, and ‘socioeconomic status’. Only articles published in English were examined. Studies were included if pain or pain symptoms were examined as either an outcome or as predictor of frail health, disability or quality of life, and if participants were 21 years or younger at the time of their cancer diagnosis and had survived at least 5 years post diagnosis. Studies that used large cohorts of survivors were examined in greater detail. The search was enhanced by incorporating key publications referenced in the included articles as well as the most recent publications in the field based on the authors’ knowledge. The main studies reporting risk factors and correlates of pain among childhood cancer survivors are summarized in Table 1 [14,15,17,30,31,32,33,34,35,36,37,38,39,40,41,42,43,44,45,46,47,48,49,50,51,52,53,54,55,56,57,58,59,60,61,62,63,64].

## 3. Pain in Childhood Cancer Survivors

### 3.1. Definition and Prevalence of Pain Among Childhood Cancer Survivors

Pain is “an unpleasant sensory and emotional experience associated with, or resembling that associated with, actual or potential tissue damage” [65]. Pain is one of the most common symptoms reported by childhood cancer survivors, with prevalence estimates ranging from 5% to 59% [14,47,53,54,62,63,66,67]. Pain among childhood cancer survivors is associated with functional limitations, restrictions in activities of daily living, and lower health-related quality of life [49,58,68,69]. A recent study, using data from the National Health Interview Survey, reported that one third of childhood cancer survivors in the United States suffer from chronic pain (i.e., pain that persists for more than three months) and that one out of six reported high-impact chronic pain accompanied by physical performance limitations and restricted daily activities [67].

Studies among childhood cancer survivors characterize pain in a variety of ways; definitions are often based either on single questions about bodily pain involving the head [44], neck, extremities, or back [47]. Questions typically qualify pain as either cancer-related or as a result of treatment [36,37]. Validated pain scales have been less frequently used [17,34], while quality of life questionnaires are often employed [15,32,33,44,56]. The majority of the studies investigating pain in childhood cancer survivors are observational and include both survivors and either peer or sibling comparison groups [14,39,40,44,46,52,55]. The percentage of survivors with pain varies by site and the method of assessment and includes migraines (15.5%) [14], other headaches (20.5–25%) [14,44], bodily pain (5.2%) [44], head (35.9%) [47,50], back/neck (23–48.5%) [51,55], and cancer- or treatment-related pain (21%) [14]. 

### 3.2. Risk Factors for Pain Among Childhood Cancer Survivors

Identified risk factors for pain among childhood cancer survivors include host, diagnostic and treatment, neuropsychological factors, and socioeconomic characteristics. Several studies [14,17,34,40,42] indicate that female survivors and those who are older at diagnosis report more intense pain when compared to males or those younger than age 10 at diagnosis. Underrepresented racial/ethnic groups, especially Hispanic and African American, are at elevated risk for pain compared to Whites [14,35,60]. Childhood cancer survivors treated for retinoblastoma (57.9%), neuroblastoma (45.7%), Wilms tumor (45.2%), soft tissue sarcoma (45.2%), and bone tumor (38.7%) report the highest prevalence of pain [14,17,70]. Treatment-related risk factors for pain include exposure to radiation, chemotherapy, and surgery [12,71]. Specifically, survivors treated with either cranial or head and neck radiation [14] are more likely than those not exposed to report headaches or migraine, and those treated with abdominal radiation are more likely than those not exposed to report poor mental health, functional impairment, and activity limitation [42]. Up to 14% of survivors of acute lymphoblastic leukemia treated with vincristine report cancer-related pain during survivorship [57], and long-term survivors treated with limb-sparing surgery or amputation are about twice more likely to experience pain with daily interference compared to those who were not treated with these surgeries [61].

In terms of neuropsychological factors, depressive symptomatology [62], higher symptoms of post-traumatic stress and maladaptive cognitive–emotional responses to painful situations (i.e., pain catastrophizing) [63] are associated with greater risk of chronic pain among long-term survivors. Lastly, survivors with lower annual household incomes, those who did not complete high school, and those who were married were more likely to report pain due to cancer compared to those with higher household incomes, those who graduated from high school, and those who were not married [14].

### 3.3. Correlates of Pain Among Childhood Cancer Survivors

The impact of pain among children with cancer and among childhood cancer survivors on future adverse health outcomes has not been evaluated. However, previous data suggest that poor emotional health, history of chronic diseases, lower socioeconomic status, and poor quality of life are correlates of pain among adult survivors of childhood cancer [45,48,59,64,72].

One investigation evaluated associations between pain and emotional health in 1863 adult survivors of childhood cancer treated at a single institution. At a median age of 32 years at follow-up, cancer-related pain was associated with higher odds of emotional distress (odds ration [OR] 8.72; 95% confidence interval [CI], 5.32–4.31) [48], and rates of emotional distress increased (4% per year) with increasing levels of self-reported pain [48].

Data from the Childhood Cancer Survivor Study (CCSS), a large multi-institutional cohort of five-year survivors, followed participants for more than 25 years with eight different cancer types diagnosed between 1970–1999. Among 10,012 of the childhood cancer survivors in this cohort, at a median age of 31 years at assessment, it was found that survivors with grade 3–4 chronic health conditions report higher odds of pain, recurrent pain, and pain interference compared to those with no or a lower severity of chronic conditions [60].

Further, a recent report from the St. Jude Lifetime Cohort Study assessed pain and multiple functional outcomes in 2836 long-term survivors of childhood cancer with a mean age of 32.2 ± 8.5 years and mean time from diagnosis of 23.7 ± 8.2 years. Survivors who endorsed moderate to severe pain with daily interference had a higher risk of neurocognitive impairment, multiple physical performance deficits, reduced social functioning, and poor physical and mental health-related quality of life [61]. Another study using 3211 survivors from the same cohort (mean age 31.2 ± 8.4 years and mean time from diagnosis 22.7 ± 8.3 years) also demonstrated that survivors with pain have a 20% higher risk to worry about relapse compared to survivors without pain [64].

Another group of investigators evaluated 116 pediatric brain tumor (PBT) survivors with a mean age 10.0 ± 4.9 and a mean time of 10.6 ± 4.75 years from diagnosis and found that survivors who reported “moderate pain” ≥ 2 days/week or “severe pain” ≥ 1 day/week also reported lower quality of life across all domains of the Pediatric Quality of Life Inventory (PedsQL) and had lower physical health summary and psychosocial health summary scores [72]. Although most of these studies are cross-sectional so that temporality cannot be determined, it appears that pain is associated with adverse outcomes among childhood cancer survivors.

## 4. Frailty in Childhood Cancer Survivors

### 4.1. Definition and Prevalence of Frailty Among Childhood Cancer Survivors

Frailty is a phenotype characterized by reduced physiologic reserve and increases vulnerability to stressors that further dysregulate multiple physiologic systems [73,74]. Frailty can be characterized both as a phenotype, where clinical measures of physiologic health are evaluated/observed, or as an accumulation of deficits [74,75]. The most commonly used phenotypic model defines frailty as the presence of three or more of poor grip strength, slow walking speed, low physical activity, exhaustion, and unintentional weight loss [74]. A prefrailty stage is identified when at least two of the above criteria are met.

Observational evidence suggests that a significant proportion of childhood cancer survivors develop frailty early in life. Frailty prevalence estimates among childhood cancer survivors range from 6.4% to 13.1% [18,19]. Frailty prevalence is higher among older survivors [19] and doubles during an average time of 5 years, with a statistically significant increase over time on each frailty component [76].

### 4.2. Risk Factors for Frailty Among Childhood Cancer Survivors

Frailty in childhood cancer survivors is linked to the host characteristics, type of cancer diagnosis, and treatment exposures [29,77]. Frailty is more common among female than male survivors [19], although differences decrease over time and are no longer significant at late follow-up [76]. Survivors of childhood bone tumors, central nervous system tumors, and Hodgkin’s lymphoma are at higher risk for frailty [19]. Treatment-related risk factors associated with frailty among childhood cancer survivors include radiation to the brain or abdomen and pelvis, extremity amputation, lung surgery, and platinum exposure [19].

### 4.3. Correlates of Frailty Among Childhood Cancer Survivors

The correlates and consequences of frailty among childhood cancer survivors include chronic health conditions, lifestyle factors, cognitive functioning, emotional distress, and low socioeconomic status [18,19,78].

In a cohort of childhood cancer survivors (n = 1922, mean age 33.6 ± 8.1 years, 50.3% male) from the St. Jude Lifetime Cohort, childhood cancer survivors classified as frail using the phenotype model were 2.2 times more likely (95% CI 1.2–4.2) to develop new onset chronic conditions over (median = 3.46; range = 1.03–4.97) the years compared to those who were non-frail [18]. Within 10,899 survivors from the CCSS aged 37.6 ± 9.4 years and at least 5 years from diagnosis, the prevalence of prefrailty and frailty was greater among those with respiratory, neurologic, musculoskeletal, cardiac, and endocrine conditions, as compared to those without organ system-specific chronic conditions [19]. In addition, both of these studies showed that the prevalence of frailty and prefrailty was higher among survivors with longer duration of chronic conditions [18,19].

Among lifestyle factors, the aforementioned CCSS study showed that current smoking, sedentary behavior, and obesity (body mass index ≥ 30.0 kg/m^2^) are associated with frailty and prefrailty in childhood cancer survivors [19].

Regarding neurocognitive functioning, Williams et al. [79] investigated cross-sectional and longitudinal associations between the frailty phenotype and cognitive functioning among 10-year adult survivors of childhood cancer from the St. Jude Lifetime Cohort (n = 845, mean age 29.7 ± 6.8 year at baseline assessment, mean 21.7 ± 7.1 years since diagnosis). Compared to non-frail survivors, frail survivors demonstrated worse performance at baseline on measures of memory and processing speed, and greater decline in the domains of memory, processing, attention and executive function over a 5-year follow-up. Fewer and weaker associations were also observed between prefrail status and cognitive functioning.

Finally, Smitherman and colleagues, using data from the University of North Carolina (UNC) Cancer Survivorship Cohort (n = 271), examined associations between patient characteristics and frailty or prefrailty among survivors of adolescent and young adult (AYA) cancers (mean age 34.5 ± 4.5 years at diagnosis, 4.5 ± 4 years at evaluation, 29% male). Survivors with comorbid depression or anxiety (prevalence ratio [PR] 2.4, 95% CI: 1.51–3.67), or whose ongoing medical care was delayed because of no health insurance (PR 2.7, 95% CI: 1.63–4.59), were more likely to be frail or prefrail [78].

## 5. Association Between Pain and Frailty in Childhood Cancer Survivors

Childhood cancer survivors are at risk for pain due to their cancer and its treatment, which may eventually predispose them to a greater risk of adverse health outcomes, comorbidities, functional impairment, disability, and frailty [15,47,80,81]. There are no studies that specifically evaluate the association between pain and frailty in childhood cancer survivors. However, existing data on the high prevalence and shared correlates of frailty and pain suggest that these associations may be particularly impactful or relevant to the health of childhood cancer survivors. Also, data from other populations suggest that pain and frailty have a bidirectional association.

### 5.1. Evidence from Non-Cancer Populations

Numerous studies investigated the relationship between pain and frailty in the geriatric population, due to the increasing prevalence of both conditions with aging. Among older adults, pain is associated with lower gait speed [82], malnutrition [83], and poor grip strength [84], all components of the frailty phenotype. It is likely that pain experienced for a prolonged period of time triggers specific stress mechanisms, diminishes physiologic reserve, perpetuates inactivity, and eventually results in frailty [85]. A study by Wade and colleagues [85] using cohort data from the European Male Ageing Study (EMAS) and including 2736 community-dwelling men with a mean age of 59.2 years ± 10.6 years reported that participants with some pain at baseline were 1.59 times (95% CI 1.00, 2.55) more likely to develop frailty than those without pain using the frailty index (FI). They also reported that participants with chronic widespread pain, defined as long-lasting pain in multiple body regions that restricted their ability to do daily activities, were 5.14 times (95% CI 2.82, 9.38) more likely to develop frailty at follow-up (mean 4.3 ± 0.3 years) compared to those with no pain. This association was significant even after adjusting for factors such as smoking status, BMI, and depressive symptoms. Another investigation by Megale and colleagues [86] used longitudinal data from the Concord Health and Ageing in Men Project (CHAMP), a prospective population-based cohort study (n = 1705) of individuals aged ≥ 70 years to examine associations between pain and frailty using the Cardiovascular Health Study (CHS) frailty phenotype criteria. They reported that participants with chronic pain were 1.60 times (95% CI: 1.02–2.51) more likely to develop frailty over 5 years of follow-up compared to those without pain. An investigation by Blyth and colleagues [87] in a sample of 1705 community dwelling persons ≥ 70 years from the CHAMP survey found a significant association between pain and frailty (OR = 1.7; 95% CI = 1.1–2.7) after adjusting for sociodemographic characteristics, comorbidities, self-reported depressed mood, and arthritis. Another study conducted among 1545 community-dwelling Mexican Americans aged ≥ 67 years from the Hispanic Established Populations for the Epidemiological Study of the Elderly (1995/96 to 2012/13) examined the association between pain and frailty and found that pain was significantly associated with higher odds of becoming frail (1.71; 95% CI: 1.41–2.09) [20]. They also reported that male sex, lower educational attainment, lower score on the Mini Mental State Examination, hip fracture, high depressive symptoms, and ADL disability were associated with higher odds of becoming frail over 18 years of follow-up [20]. Using a population-based cohort of Spanish community-dwelling individuals aged ≥ 60 years, Rodríguez-Sánchez et al. found that individuals with more frequent and more intense pain and a greater number of pain locations were increasingly more likely to become frail after 3 years, suggesting a positive dose–response relationship between pain and frailty risk. These associations were significant after adjusting for sociodemographic and health behaviors (i.e., smoking, alcohol use, screen time, and adherence to Mediterranean diet) [88]. Overall, a recent meta-analysis of five prospective European studies with over 13,100 participants (54% men, mean age between 59 and 85 years) estimated that participants with persistent pain at baseline had doubled risk of developing frailty during the follow-up period (pooled relative risk [RR] = 2.22, 95% CI = 1.14–4.29) [89].

Despite the origin of pain, the pain experience triggers a complex interaction of neural and molecular pathways designed to modulate both the physical and emotional experiences of pain. Acutely, these responses are protective [90]. However, over or persistent stimulation of these control mechanisms can result in lasting dysfunction. A study conducted by McBeth and colleagues [91] among 241 community-dwelling individuals (mean age 47.3 ± 3.2 years, 37% male) with no baseline chronic widespread pain measured serum cortisol (a measure of hypothalamic–pituitary–adrenal (HPA) axis function) in response to a stressor (a pain-threshold examination) and to dexamethasone administration and found that those with abnormal levels were predisposed to new-onset chronic widespread pain after a follow-up of 15 months. Another study by Leng and colleagues [92] conducted among 558 women aged 65 to 101 from the Women’s Health and Aging Study (WHAS) I and 548 women aged 70 to 79 from both the WHAS I and II examined the association between white blood cell (WBC) count and interleukin-6 (IL-6) and prevalent frailty. They found that the higher WBC count and IL-6 levels, both markers of inflammation, were independently associated with prevalent frailty in community-dwelling older women [92].

Besides hormonal dysregulation and systemic inflammation, biobehavioral mechanisms have been proposed whereby persistent pain leads to physiologic frailty through reduced mobility and resting energy expenditure, inadequate macronutrient intake, depression, and increased social isolation [93]. Nonetheless, bidirectional mechanisms are also plausible. For example, frailty may lead to the onset of neurologic, endocrine, and musculoskeletal conditions that affect the central and peripheral nociceptive systems, resulting in altered perception of pain [89,94]. In addition, as postulated by the fear-avoidance model [95,96], the cognitive interpretation of pain as a threatening experience along with negative affect may result in long-term muscular disuse and disability, perpetuating the vicious cycle of chronic pain.

### 5.2. Possible Mechanism in Childhood Cancer Survivors

Although studies have not yet examined associations between pain and frailty in childhood cancer survivors, reports that pain and frailty are both prevalent in this population, and that pain and frailty are associated in other populations with an aging phenotype, suggest this association is highly likely in this vulnerable group of individuals.

In aging adults, it is hypothesized that pain leads to frailty through the mechanism of “pain homeostenosis” [94], whereby persisting pain simultaneously impacts multiple physiologic systems, reduces reserves and homeostatic capacity, and leads to enhanced susceptibility to stressors that can accelerate or precipitate frailty [97]. Biological changes in the brain as a result of frailty onset are hypothesized to inhibit descending pain modulatory systems [94,98], thus contributing to the development of new onset or exacerbating existing pain presentations. Disruptions in HPA axis function may be included in these mechanisms, where persistent pain is understood to increase cortisol [99], and cortisol increases are further associated with frailty onset [100]. Inflammatory immune responses are related in instances of both chronic pain and frailty, notably in measures of C-Reactive protein and interleukin-6 [101,102,103], implicative of potentially bidirectional mechanistic pathways for both phenomena. Preclinical research has further identified pathophysiological mechanisms by which pain and frailty may develop and persist. These include alterations in pro- and anti-inflammatory markers, cytokine release, repair of damaged DNA, microglial activation, increases in oxidative stress, hormonal fluctuations, and changes in brain-derived neurotropic factor (BDNF) levels [104,105,106,107,108,109]. Despite these strong mechanistic overlaps between chronic pain and phenotypic frailty, there is yet to be a direct underlying pathway which is understood to link the two entities.

Within a bio-psycho-social model of care, other mechanisms could explain the link between pain and frailty. At a psychological level, the fear-avoidance model postulates that distorted and exaggerated cognitive–emotional responses to current and future painful sensory experiences (i.e., pain catastrophizing) lead to fear of pain and avoidance behaviors, resulting in reduced physical activity, loss of physical function, and increased disability, which characterize the frailty phenotype. Among older adults with chronic pain, catastrophizing [110] and fear of movement [111,112] (influenced by anxiety, depression, and executive dysfunction [113,114]) predict subsequent physical inactivity and disability. At the societal level, pain may lead to frail status through interpersonal relationships [115], socioeconomic position [116,117], and cultural constructs [118].

**Figure 1:**
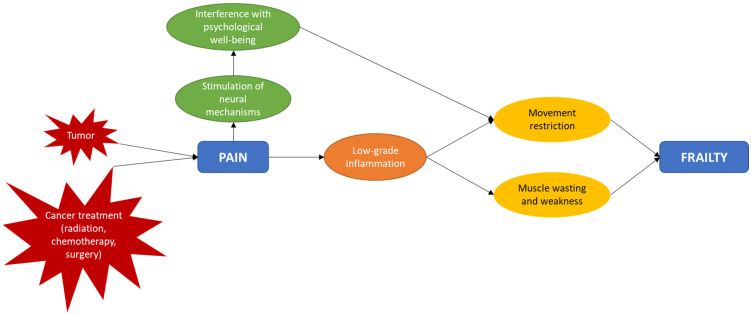
Mechanism of proposed pathway of association between pain and frailty among childhood cancer survivors, considered within a survivor’s unique social and cultural context.

For childhood cancer survivors, we hypothesize that pain, triggered acutely by cancer and its treatment, and perpetuated/maintained by maladaptive (cognitive, emotional, and behavioral) coping strategies, and dysfunctional physiologic mechanisms, contributes to the frailty phenotype, not only by interfering with movement and propagating muscle wasting and weakness, but also by stimulating neural mechanisms that interfere with psychological well-being and slow movement. Pain also triggers molecular pathways that are associated with chronic low-grade inflammation and ageing (Figure 1) [119].

## 6. Interventions to Prevent or Remediate Cancer-Related Pain and Frailty

Given the high prevalence and significant impact of pain on functional outcomes among childhood cancer survivors, it is vital to support survivors to achieve optimal pain management. Failure to do so may result in engagement in maladaptive coping strategies, including substance misuse and/or abuse [120]. Evidence shows significantly higher prevalence of potential misuse of prescription opioids or substance use disorder within 1 year after therapy among children (1.4% vs. 0.1%), adolescents (4.7% vs. 1.4%), and young adults (9.4% vs. 4.3%) who survived childhood cancer, and the risk of engaging in theses problematic patterns remains significantly elevated even after adjusting for sociodemographic factors and health status. Alarmingly, relief of physical pain (64%) and help with emotional problems (7%) are the most common reasons for misuse of prescription opioids among adolescent and young adult survivors, suggesting inadequate pharmacological management of pain and mental health support [120]. Among long-term adult survivors, persistent/increased pain and anxiety are associated with 7.7-fold and 2.6-fold increased odds of subsequent opioid use, and persistent/increased depression is associated with 2.6-fold increased odds of subsequent marijuana use [121].

Because cancer-related pain can have both physical and psychosocial origins, multimodal interventions are likely to be the most effective and should include pharmacological and non-pharmacological approaches [122,123]. Pharmacological approaches for pain management in adult cancer survivors follow the three-level ladder proposed by the World Health Organization, proposing progressive escalation from non-opioid analgesics to weak and finally strong opioids [124,125,126]. However, it should be noted that, among adult survivors of childhood cancer, analgesics and antidepressant medications are associated with *worsened* pain and pain interference with daily activities over time, suggesting that pharmacological interventions alone may be ineffective or even harmful in this population [60]. Non-pharmacological approaches targeting sensory, cognitive and emotional aspects of pain include physical therapy, occupational therapy, acupuncture, regular exercise, psychosocial interventions, behavioral therapy, music therapy, massage therapy, and complementary and alternative therapies [127,128,129,130,131]. Combinations of these approaches, which can also be implemented in groups to address social aspects of pain (e.g., social support), are available in many pain management programs [127,128,129,130,131]. Survivors of childhood cancer may be interested in these non-pharmacological interventions, as previous research showed that pain is associated with 50–90% increased odds of using complementary and alternative therapies in conjunction with conventional medicine in this population [36]. Holistic approaches are important for patients and survivors with poorly managed chronic pain, whose discomfort is exacerbated not only by internal, but also by external, stressors [38,129]. A framework of general interventions to remediate cancer-related pain in childhood cancer survivors is described in Table 2.

Conversely, to date, no studies have been conducted to prevent or remediate frailty in childhood cancer survivors. Nonetheless, pharmaceutical and non-pharmaceutical approaches that have shown to improve biomarkers of ageing in older adults hold promise as frailty interventions for childhood cancer survivors [28]. Pharmaceutical approaches include agents that mimic caloric restriction, reduce cellular senescence, and promote genomic stability and mitochondrial functioning [28]. A clinical trial is currently ongoing to test the efficacy, safety, and tolerability of pharmaceutical regimens targeting senescent cells. Non-pharmacological interventions for frailty tested in the geriatric population include physical activity, nutrition, psychosocial or cognitive training, and multifactorial, geriatric comprehensive assessments. In a recent meta-analysis, including 21 trials and eight intervention types, physical activity (with or without nutritional supplements) was found to be the most effective intervention to prevent or treat frailty [137].

Given the high prevalence of pain and frailty among childhood cancer survivors and their common risk factors and functional correlates, the possibility to develop future interventions that effectively target both pain and frailty warrants further attention. For example, in addition to beneficial effects on physiologic frailty, physical activity may improve pain through the activation of the endogenous opioid system, weight loss and subsequent reduced joint pressure, improved metabolism, and increased resistance of the musculoskeletal system [138]. In addition, dietary interventions were found to be beneficial for the management of non-cancer pain conditions using anti-inflammatory and antioxidant agents [139,140,141], although this line of research is still limited for cancer-related pain [142]. Because healthy/unhealthy behaviors are often clustered rather than isolated [143,144,145], interventions promoting multifaceted lifestyle change [146] may have greater potential to address both pain and frailty among childhood cancer survivors. This population is likely unique given their past exposures to chemotherapy, radiation, and surgery, and the impact of these treatments on processes of physical and psychological development. More research is needed to understand the associations between pain and frailty and to identify the best approach for treating pain and frailty in childhood cancer survivors.

## 7. Conclusions

This review highlights an important gap in the literature regarding the potential associations between pain and frailty among childhood cancer survivors. Some strengths and limitations should be considered. Given its narrative nature, this review does not provide a thorough and systematic evaluation of rigorously selected articles about pain and frailty among childhood cancer survivors. Nonetheless, it offers a comprehensive overview of the existing literature about each adverse health outcome, with emphasis on more robust evidence from large epidemiological studies. Despite the similar prevalence of frailty among survivors of childhood cancer in their 30s and older adults without cancer in their 60s [18], different mechanisms may govern the associations between pain and frailty in these populations. These mechanisms may involve the unique biological impact of cancer history and treatment exposures on the ageing process and pain etiology as well as distinctive psychosocial copying strategies used to deal with personal and societal expectations of an *atypical* deterioration of health and failure to fully attain age-appropriate developmental milestones (as opposed to a *typical* age-related health decline). In the cancer context, due to increasing cancer incidence with age, frailty research has focused primarily on older adults with cancer [147], but research has shown an additive effect of aging and cancer on their functional decline [148,149]. In comparison, fewer studies have examined frailty among middle-aged adults with cancer, but these studies are limited by small samples [150] or specific cancer diagnoses (e.g., colorectal or breast cancer) that restrict generalizability to childhood cancer survivors. Although the general geriatric population represented the best model for our review, future research and interventions should fully embrace a bio-psycho-social model of care to comprehensively consider the multidimensional relationship between pain and frailty among childhood cancer survivors across the ageing continuum.

In summary, childhood cancer survivors are at risk for experiencing both pain and frailty as they age. However, data are limited describing the associations between these two long-term adverse health outcomes. Information from other populations suggests potential synergistic effects of pain and frailty in childhood cancer survivors. Understanding potential associations between pain and frailty and the various confounders of this association has the potential to inform interventions designed to prevent the onset of frail health and improve pain-related disability in childhood cancer survivors. Multidisciplinary interventions are needed to prevent/delay adverse health outcomes, including pain and frailty, among childhood cancer survivors as they progress through life, allowing them to maintain independence and quality of life over time.

## Figures and Tables

**Table 1:** Main articles reporting risk factors and correlates of pain among childhood cancer survivors.

Author, Year	Study Population (N)	Diagnosis	Age at Study, in Years Mean (SD/Range)/ N (%)	Age at Diagnosis, in Years Mean (SD/Range)/ N (%)	Time Since Diagnosis, in Years Mean (SD/Range)/ N (%)	Pain Measure Used	Pain-Related Findings
Hudson et al., 2003 [30]	9535 survivors; 2916 sibling controls M = 5083 (53.3%) F = 4452 (46.7%)	Multiple	26.8 (6.2)	10.0 (5.6)	17.4 (4.6)	Pain associated with cancer or its treatment varies from 1 to 5, with 1 = no pain and 5 = severe excruciating pain.	Pain prevalence reported: 8.5% (18–24 yr), 9.7% (25–29 yr), 12.8% (30–34 yr and ≥35 yr). Pain prevalence increases by age of diagnosis: 8.5% (0–4 yr), 8.6% (5–9 yr), 11.1% (10–14 yr), 12.9% (15–21 yr). Highest pain prevalence was in survivors of sarcoma (14.8%) and bone cancer (23%).
Langeveld et al., 2004 [31]	400 survivors; 560 controls M = 220 (55%) F = 180 (45%)	Multiple	24 (4.9)	8 (4.6)	16 (5.6)	Medical Outcome Study Scale (MOS-24)	Pain was reported less in survivors compared to the controls.
Meeske et al., 2005 [32]	161 M = 74 (46%) F = 87 (54%)	ALL	18–41	7.4 (0–18)	13.9 (4–23)	SF-36 and scale measuring bodily pain.	Pain was associated with fatigue and depression and risk increased over time.
Maunsell et al., 2006 [33]	1334 survivors; 1477 controls M = 641 (48.1%) F = 693 (52.0%)	Multiple	23.0 (5.2)	0–4 yr: 326 (24.4%) 5–9 yr: 275 (20.6%) 10–14 yr: 196 (14.7%) 15–19 yr: 537 (40.3%)	5–19	SF-36 and scale measuring bodily pain.	No differences in pain among survivors and controls by gender; higher pain was reported among bone cancer survivors and those with organ dysfunction as compared to controls.
Pogany et al., 2006 [34]	2152 survivors; 2432 population-based controls M = 1101 (51.2%) F = 1051 (48.8%)	Multiple	5–12 yr: 412 (19.1%) 13–15 yr: 381 (17.1%) 16–19 yr: 468 (21.8%) 20–24 yr: 383 (17.8%) 25–29 yr: 335 (15.6%) 30–37 yr: 173 (8.0%)	<1 yr: 195 (9.1%) 1–4 yr: 768 (35.7%) 5–9 yr: 456 (21.2%) 10–14 yr: 356 (16.5%) 15–19 yr: 377 (17.5%)	≥5	Health Utilities Index questionnaire (HUI 3).	Pain prevalence in survivors (40.3%) and controls (44%); higher pain was seen in survivors diagnosed at older age and those with bone cancer; females had higher pain compared to males.
Punyko et al., 2007 [35]	417 survivors; 2865 siblings as controls M = 237 (57%) F = 180 (43%)	Primary rhabdomyosarcoma	18–24 yr: 176 (42%) 25–34 yr: 198 (47%) 35–45 yr: 43 (10%)	<1 yr: 22 (5%) 1–4 yr: 119 (29%) 5–9 yr: 113 (27%) 10–14 yr: 94 (23%) 15+ yr: 69 (17%)	18.0 (7.3–28.8)	Cancer-related pain measured as dichotomous variable: “medium amount of pain/a lot of pain/very bad, excruciating pain” or “no pain/small amount of pain”.	Higher prevalence of cancer-related pain seen in ever married survivors and survivors with a lower rate of high school completion.
Alessi et al., 2007 [17]	644 M = 349 (54.2%) F = 295 (45.8%)	Multiple	≥15	n/a	≥5	HUI 2/3	Prevalence of pain reported higher among female than male survivors. Higher risk of pain among survivors < 10 years of age compared to survivors > 10–14 years of age at diagnosis. Higher pain in CNS cancer, retinoblastoma and bone tumor survivors than others.
Ness et al., 2008 [15]	7147 M = 3481 (48.7%) F = 3666 (51.3%)	Multiple	≥18	10 (5.6)	≥5	SF-36 and scale measuring bodily pain.	Higher odds of bodily pain were reported among survivors with poor physical performance, emotional health, and lower executive functioning.
Mertens et al., 2008 [36]	9984 survivors; 2474 siblings as controls M = 5166 (51.7%) F = 4818 (48.3%)	Multiple	<18 yr: 622 (6.23%) 18–29 yr: 4925 (49.33%) 30–39 yr: 3504 (35.10%) ≥40 yr: 933 (9.34%)	0–4 yr: 4060 (40.7%) 5–9 yr: 2190 (21.9%) 10–14-yr: 2024 (20.3%) 15–20 yr: 1710 (17.1%)	≥5	Self-reported pain (Y/N): i. any pain due to cancer or treatment related, ii. migraines or headaches, iii. use of pain medication/muscle relaxant, iv. prolonged pain/abnormal sensations.	Survivors who reported any pain have greater use of complementary and alternative medicine than those with no pain.
Cox et al., 2009 [37]	838 M = 385 (45.9%) F = 453 (54.1%)	Multiple	30.9 (7.5)	9.2 (5.8)	21.7 (4.5)	Pain associated with cancer or its treatment varies from 1 to 5, with 1 = no pain and 5 = severe excruciating pain.	Reduced cancer-related pain associated with higher stamina; higher cancer-related pain associated with increased anxiety, fatigue, and future health fears.
Ishida et al., 2010 [38]	185 survivors; 1000 controls M = 77 (41.6%) F = 108 (58.4%)	Multiple	M: 23.1 (5.1) F: 23.2 (4.9)	M: 8.5 (5.0) F: 8.3 (4.8)	≥5	Chronic pain was measured as single item.	Greater risk of chronic pain was reported in survivors who received, and did not receive, stem cell transplants than in the general population.
Recklitis et al., 2010 [39]	9126 survivors; 2968 sibling controls M = 4814 (52.8%) F = 4312 (47.3%)	Multiple	≥18	0–20	≥5	Cancer-related pain, measured as “none/small amount”, “medium amount”, or “a lot/very bad”.	Survivors experiencing suicidal ideation reported higher prevalence and severity of cancer-related pain; suicidal ideation associated with greater use of pain medication among survivors and controls.
Lu et al., 2011 [14]	10,397 survivors; 3034 siblings as controls M = 5593 (53.8%) F = 4804 (46.2%)	Multiple	≥18	0–3 yr: 1899 (18.3%) 4–10 yr: 3607 (34.7%) 11–14 yr: 2398 (23.1%) 15–20 yr: 2493 (24.0%)	16.5 (4.9)	Survivors were asked if they had ever been told by a doctor/other healthcare professional that they have, or have had, any of the following pain conditions: “prolonged pain or abnormal sensation in the arms, legs, or back”, “migraine”, or “other frequent headaches. “Participants were given response options of “yes”, “no”, or “not sure”.	Frequency of pain/abnormal sensation (12.3%), migraines (15.5%), other headaches (20.5%) was reported. Pain (21%) was cancer and treatment related, and greater risk of pain was reported among survivors than controls. Prevalence of pain conditions higher among females, those with lower educational attainment, minority status, unemployed, and those who were single. Higher pain was reported among those with younger age at diagnosis, history of non-Hodgkin lymphoma, Wilms tumor, or neuroblastoma. Greater odds of pain medications use were reported among those with soft tissue sarcoma and bone cancer compared to leukemia.
Bowers et al., 2012 [40]	99 survivors; 53 siblings as controls M = 56 (56.6%) F = 43 (43.4%)	ALL	18.1 (3.34)	≤18	≥5	Single items assessing self-reported back pain and hip pain (Y/N).	Higher prevalence of back pain was reported among survivors (44.4%) as compared to siblings (21.2%); female survivors reported higher risk of pain as compared to male survivors and female siblings.
Essig et al., 2012 [41]	457 survivors M = 229 (50.1%) F = 228 (49.9%)	ALL	16–24.9 yr: 236 (51.6%) 25–29.9 yr: 110 (24.1%) 30–34.9 yr: 62 (13.6%) ≥35 yr: 49 (10.7%)	0–4.9 yr: 227 (49.7%) 5–9.9 yr: 136 (29.8%) ≥10 yr: 94 (20.6%)	≥5	SF-36, bodily pain scale.	ALL survivors who relapsed reported higher rates of bodily pain as compared with ALL survivors with no relapse.
Marina et al., 2013 [42]	1094 M = 539 (49.3%) F = 555 (50.7%)	Childhood extremity (upper and lower) sarcoma survivors	18 (5–25)	13 (0–20)	5–9 yr: 98 (9.0%) 10–14 yr: 362 (33.1%) 15–19 yr: 369 (33.7%) 20–24 yr: 231 (21.1%) 25–29 yr: 34 (3.1%)	Measure: pain due to cancer or its treatment, rated as “none/small amount”, “medium amount”, or “a lot/very bad”.	Older age, being female, and history of abdominal radiation were associated with pain.
Boman et al., 2013 [43]	528 survivors M = 274 (51.9%) F = 254 (48.1%)	CNS tumors	26.3 (4.9)	10.5 (4.4)	15.7 (5.03)	HUI 2/3 Health status and HRQOL, including pain.	Pain was associated with poor self-perception outcomes, including body image, and lowered sports/physical activities-related confidence.
Brinkman et al., 2013 [44]	7080 survivors; 384 siblings as controls M = 3468 (49%) F = 3612 (51%)	Multiple	31.6 (7.5)	7.9 (5.9)	23.6 (4.54)	Measure: bodily pain, headache pain. Not clear how pain was assessed.	Pain more commonly reported in survivors than siblings (*p* < 0.001). Headache pain: 25.0% survivors and 20.1% siblings. Other bodily pain: 5.2% survivors and 2.1% siblings. No pain: 69.6% survivors and 77.6% siblings
Brinkman et al., 2013 [45]	10,378 survivors; 3206 siblings as controls M = 5582 (53.8%) F = 4796 (46.2%)	Multiple	18–24 yr: 4386 (42.3%) 25–29 yr: 2761 (26.6%) 30–34 yr: 1976 (19.0%) ≥35 yr: 1255 (12.1%)	0–4 yr: 2536 (24.4%) 5–9 yr: 2487 (24.0%) 10–14 yr: 2868 (27.6%) 15–19 yr: 2202 (21.2%) ≥20 yr: 285 (2.8%)	5–10 yr: 1026 (9.9%) 11–15 yr: 3042 (29.3%) 16–20 yr: 3630 (35.0%) ≥21 yr: 2680 (25.8%)	SF-36 (bodily pain subscale), past 4-week pain frequency and extent to which pain interferes with normal activities. Pain questionnaire (CCSS survey). Survivors were asked if they had “ever been told by a doctor/other health care professional that they have or have had any of the following pain conditions”: “prolonged pain or abnormal sensation in the arms, legs, or back”, “migraine”, or “other frequent headaches”. Participants were given response options of “yes”, “no”, or “not sure”.	1. Survivors were significantly more likely to report pain than their siblings. 2. Pain symptoms were associated with the use of medications for psychiatric conditions (e.g., depression and anxiety). 3. Headache (vs. none) and bodily pain (vs. none) predicted psychoactive medication use at baseline and new onset psychoactive medication uses in survivors. 4. Reduced functioning on the HRQOL Pain Subscale was associated with psychoactive medication (non-opioids, opioids, antidepressants, and muscle relaxants).
Brinkman et al., 2013 [46]	4569 M = 2227 (48.7%) F = 2342 (51.3%)	Multiple	27.4 (6.0)	10.0 (5.6)	17.4 (4.6)	Pain due to cancer or cancer treatment classified as none/small amount, medium amount, or a lot/very bad.	Pain severity associated with emotional distress and severity. Decreased anxiety associated with reduced risk of cancer related pain. Symptoms of somatization were associated with increased cancer-related pain over time.
Huang et al., 2013 [47]	1667 M = 809 (48.5%) F = 858 (51.5%)	Multiple	33.7 (8.2)	n/a	25.5 (7.8)	Pain ratings were measured in head, neck, and back, and pain involving sites other than the head, neck, and back.	Prevalence of pain was reported as 58.7% in regions apart from head and neck, 35.9% in the head, 48.5% in the back/neck. Pain involving sites other than head, neck, and back was associated with poor HRQOLs.
Oancea et al., 2014 [48]	1863 M = 928 (49.8%) F = 935 (50.2%)	Multiple	32 (26–38)	median 7	≥10	Cancer-related pain, rated as “no pain”, “small amount of pain”, “medium amount”, “a lot of pain” or “very bad, excruciating pain”.	Cancer-related pain was associated with higher emotional distress, anxiety, depression, and somatization.
Zeller et al., 2014 [49]	27 survivor cases with PCF, 35 survivor controls without PCF	ALL or Lymphoma	PCF: 33.7 (6.6) No PCF: 34.4 (7.3)	10.1 (1.6–18.4)	25.3 (11.3–39.9)	Brief Pain Inventory (BPI), Pain severity and interference. Algometer Commander, J-Tech Medical (measure of pain sensitivity).	Survivors with Persistent Chronic Fatigue (PCF) had higher pain prevalence, higher pain severity, and reported increased interference with their function. Most common pain location reported was neck and shoulder.
Zeller et al., 2014 [50]	35 survivor cases with CF; 52 survivor controls without CF	ALL or Lymphoma	33.0 (20.5–53.1)	n/a	25.2 (11.3–39.9)	Self-report measure of headache, muscular pain, and joint pain rated on a scale from 1 (never/rarely present) to 5 (present at all time).	CF survivors had higher rates of headache, muscular pain, and joint pain as compared to the controls.
Khan et al., 2014 [51]	162 M = 90 (56%) F = 72 (44%)	ALL	15.7 (6.9–29)	3.9 (0.4–18.6)	10.2 (5–22.7)	Back pain was assessed using the clinical questionnaires (Y/N). The Modified Hanover Low Back Pain Disability Questionnaire was used to assess the disability.	Back pain prevalence was reported as 23% among survivors.
Ozono et al., 2014 [52]	185 survivors; 72 siblings as controls; 1000 population controls M = 77 (41%) F = 108 (59%)	Multiple	23.6 (4.6)	8.3 (4.8)	15.3 (5.8)	Single “chronic pain” item measured.	Higher rates of chronic pain were reported in survivors as compared to population controls.
Schultz et al., 2014 [53]	180 survivors M = 85 (47%) F = 95 (53%)	AML	20 (8–39)	4 (0–20)	13.5 (6–22)	Cancer related pain (Y/N)	Prevalence of pain was 5%. No differences were reported among those with cancer-related pain who received chemotherapy only and those who had received both chemotherapy and bone marrow transplant.
Phillips et al., 2015 [54]	324,396 survivors estimated based on SEER data and CCSS sample M = 161,158 (49.7%) F = 163,237 (50.3%)	Multiple	≤19	n/a	>5	Measure: Pain refers to prevalence Health status domains (CCSS survey). Current cancer-related pain, rated as “none/small amount”, “medium amount”, or “a lot/very bad”.	Prevalence estimates of pain morbidity (12%). Pain increased with age, ranging from 10% (20–29 yr) to 12% (30–39 yr) and 15% (40–49 yr).
D’Agostino et al., 2016 [55]	16,079 survivors; 3085 siblings as controls M = 8323 (51.8%) F = 7756 (48.2%)	Multiple	27.1 (5.9)	9.4 (5.6)	17.7 (4.3)	Pain questionnaire (CCSS survey): survivors were asked if they had “ever been told by a doctor/other healthcare professional that they have or have had any of the following pain conditions”: “prolonged pain or abnormal sensation in the arms, legs, or back”, “migraine”, or “other frequent headaches”. Participants were given response options of “yes”, “no”, or “not sure”.	Survivors had higher pain compared to their siblings; bodily pain and headache were associated with comorbid distress.
Huang et al., 2017 [56]	7103 survivors; 390 siblings as controls M = 3388 (47.7%) F = 3715 (52.3%)	Multiple	31.8 (7.5)	<21	mean 32	SF-36 (bodily pain subscale), past 4-week pain frequency and the extent to which pain interferes with normal activities.	Survivors with increased symptoms of anxiety, depression, and somatization reported higher pain (HRQOL) as compared to those with less emotional distress; significant association was reported between emotional distress and bodily pain.
Ness et al., 2017 [57]	14,566 survivors; 3149 siblings as controls	Multiple	70s: 28.5 (6.4) 80s: 26.9 (6.0) 90s: 25.7 (5.8) (based on treatment decades)	70s: 8.7 (5.8) 80s: 10.1 (5.8) 90s: 9.0 (6.1) (based on treatment decades)	70s: 20.3 (3.0) 80s: 17.0 (5.6) 90s: 16.8 (3.5) (based on treatment decades)	Cancer-related pain, rated as “none/small amount of pain”, “medium amount”, or “a lot/very bad”.	The percentage of survivors of ALL and osteosarcoma who reported cancer-related pain increased across treatment period (1970–1990).
Nayiager et al., 2017 [58]	75 M = 41 (54.7%) F = 34 (45.3%)	ALL	21.5 (13.5–38)	n/a	15 (10–26)	HUI 2/3	Some of the survivors who had pain reported moderate to severe disability.
Rach et al., 2017 [59]	751 M = 372 (49.5%) F = 379 (50.5%)	Hodgkin’s lymphoma	18–29 yr: 53 (7.1%) 30–34 yr: 154 (20.5%) ≥35 yr: 544 (72.4%)	0–10 yr: 150 (20%) 11–15 yr: 319 (42.5%) 16–20 yr: 282 (37.5%)	≥5	SF-36 (bodily pain subscale); cancer-related pain, rated as “none/small amount of pain”, “medium amount”, or “a lot/very bad”.	Survivors with higher bodily pain reported greater risk of fatigue and poorer sleep quality; higher cancer-related pain were associated with poor sleep quality.
Karlson et al., 2020 [60]	10,012 survivors 3173 siblings M = 5139 (51.3%) F = 4873 (48.7%)	Multiple	31 (17–57)	6.7 (0–20.99)	23 (15–35)	SF-36 (bodily pain subscale), past 4-week pain intensity and the extent to which pain interferes with normal activities.	The prevalence of moderate to severe pain, moderate to extreme pain interference, and moderate to severe recurrent pain among survivors (29%, 20% and 9%) was higher than siblings. Female sex, diagnosis of sarcoma/bone tumor, and severe/life-threatening chronic medical conditions were associated with recurrent pain. Depression and anxiety were associated with greater risk of all pain outcomes. Vitality mediated the effect of anxiety on pain and pain interference.
Tonning Olsson et al., 2021 [61]	2836 survivors 343 controls M = 1403 (49.5%) F = 1433 (50.5%)	Multiple	32.2 (8.5)	8.6 (5.6)	23.7 (8.2)	SF-36 (bodily pain subscale), past 4-week pain intensity and the extent to which pain interferes with normal activities. Pain questionnaire: survivors were asked if they had “ever been told by a doctor/other healthcare professional that they have or have had any of the following pain conditions”: “migraine”, or “other frequent headaches”. Participants were given response options of “no”, “yes, and the condition is still present”, and “yes, but the condition is no longer present”.	Severe and life-threatening chronic conditions were associated with higher risk of pain with interference. Pain with daily interference conferred greater risk of impaired neurocognition, physical functioning, social functioning and HRQOL.
Patton et al. 2021 [62]	299 survivors M = 157 (52.51%) Female: 142 (47.49%)	Multiple	5–9 yr: 32 (10.7%) 10–14 yr: 74 (24.7%) 15–19 yr: 82 (27.4%) 20–24 yr: 67 (22.4%) 25–29 yr: 31 (10.4%) 30–34 yr: 11 (3.7%) 35–40 yr: 2 (0.7%)	4.7 (0–20.1)	>2	Long-Term Survivor Questionnaire: survivors were asked the question “Since your last visit have you had any of the conditions below: (a) frequent headaches; (b) chest pain; (c) back pain and (d) any other chronic pain”. Response options for each item included “yes” or “no”.	Pain in at least one survivorship clinic visit was reported by 47% of survivors. Headache was the most frequent pain type (26.4%). Survivors of Wilms Tumor (51.5%) and Ewing’s sarcoma (50%) had the highest prevalence of pain.
Patton et al., 2021 [63]	140 survivors M = 68 (48.6%) F = 72 (51.4%)	Multiple	17.2 (4.9)	6.26 (4.90)	>5	Pain Questionnaire: self-reported measure of pain frequency, location, duration, average pain intensity in the past week, and distress due to pain. Participants were considered to have chronic pain if they are currently experiencing pain that has lasted at least 3 months or longer.	Greater posttraumatic stress symptoms, older age at evaluation, and more pain catastrophizing were significantly associated with the presence of chronic pain.
McDonnel et al., 2021 [64]	3211 M = 1652 (51.4%) F = 1559 (48.6%)	Multiple	31.2 (8.4)	8.4 (5.6)	22.8 (8.3)	SF-36 (bodily pain subscale), past 4-week pain intensity and the extent to which pain interferes with normal activities	Pain was associated with increased risk of worry about relapse. Both pain and pain interference were associated with increased risk of worry about physical problems related to cancer.

Abbreviations: ALL, Acute lymphoblastic leukemia; AML, Acute myeloid leukemia; BPI, Brief Pain Inventory; CCSS, Childhood Cancer Survivor Study; CF, chronic fatigue; CNS, central nervous system; F, female; HRQOL, health related quality of life; HUI 2/3, Health Utilities Index questionnaire; M, male; MOS, Medical Outcome Study Scale; n/a, not available; PCF, Persistent chronic fatigue; RT, radiation therapy; SD, standard deviation; SEER, Surveillance, Epidemiology, and End Results; SF-36 = Short Form (36) Health Survey; Yr, year.

**Table 2:** Intervention to remediate cancer-related pain in survivors.

Approach/Intervention	Description
Pharmacological approach	
Pain medications	For acute pain—pain medicines (opioids), analgesics, antidepressants, acetaminophen, or non-steroid anti-inflammatory drugs (NSAIDs) are widely used for treating pain in pediatric cancer [132]
	For moderate to severe pain—opioids such as codeine, oxycodone, hydrocodone, morphine, and gabapentin (nerve-related pain) are administered [126]
Non-pharmacological approach	
Physical activity	Activities including brisk walking, biking, or yoga help in improving blood circulation, strengthening muscles, and increasing joint range of motion, which helps in reducing pain-related symptoms [133,134]
Massage therapy	Body massage is effective in improving blood flow and reducing muscle spasms and stiffness, and it stretches soft tissues and relaxes the body [131]
Physical therapy	Hot packs, cryotherapy, physical exercises, electrothermal modalities, laser, traction, and compression therapy are used to improve tissue healing and reduce pain [135].
Cognitive–behavioral therapy	Group cognitive therapy (4 weeks) is effective in improving overall cognitive function, visuospatial/constructional performance, and memory (immediate and delayed) and reducing psychosocial distress [136].
Mind–body intervention	Interventions including relaxation, imagery/hypnosis, meditation, music, and virtual reality have shown improvements in pain related to cancer [127].
Transcutaneous electrical nerve stimulation (TENS)	Cancer patients treated with TENS reported improvement in pain symptoms and overall quality of life [129,130]
